# Prospecting for an HIV vaccine

**DOI:** 10.1186/s40794-017-0050-4

**Published:** 2017-03-24

**Authors:** D. M. Brett-Major, T. A. Crowell, N. L. Michael

**Affiliations:** 1 U.S. Military HIV Research Program, Walter Reed Army Institute of Research, Silver Spring, MD USA; 2 Henry M. Jackson Foundation for the Advancement of Military Medicine, Bethesda, MD USA

**Keywords:** Human immunodeficiency virus vaccine protective efficacy immunology review

## Abstract

Human immunodeficiency virus (HIV) sets several challenges for the development of a preventative HIV vaccine. Predictable, protective natural immunity against HIV does not occur and so unlike most other diseases for which vaccines exist, there are few guideposts from natural infection. Nonetheless, six vaccine efficacy trials have occurred. One in particular, the Thai trial called RV144, showed partial protective efficacy and potential ways ahead to a better vaccine approach. This coupled with other lessons from studies of acute infections as well as an increasingly complex knowledge of HIV-related vaccine immunology bring hope that a vaccine solution might be reached for this pervasive and deadly pandemic.

## Background

Human immunodeficiency virus (HIV) disease remains one of the greatest threats to global public health. According to the World Health Organization (WHO), in 2014 over one million people died from HIV, nearly thirty-seven million people had chronic infection and two million people newly acquired infections [1]. Of those persons known to be HIV infected, only 35% receive therapy from an already resource stressed global sourcing network [2]. Access to care and available prevention strategies from the U.S. President’s Emergency Program for AIDS Relief (PEPFAR) and The Global Fund are Herculean efforts to blunt the impact of the HIV pandemic, but remain insufficient to prevent the mounting HIV case burden that continues to accrue for a disease that lacks a cure and is almost universally fatal in the absence of lifelong therapy. In economic modeling of control of the HIV pandemic using competing examples of different kinds of HIV epidemics, considering a broad range of conventional and novel control approaches, the single most important intervention remained an effective vaccine [3]. The world needs an HIV vaccine.

## Why not already

A reasonable person new to the global conversation about HIV might ask, if an HIV vaccine is so critical and the pandemic known for three decades, why do we not already have an HIV vaccine? There is no simple answer to this question, though an easy one is that people do not develop natural, protective immunity to HIV infection and disease. With the notable exception of rabies, U.S.-licensed vaccines only exist for diseases for which natural immunity exists. While there are a very small group of what is known as elite controllers—people who remain HIV infected but do not develop increasing viral loads and clinical disease for long periods—the vast majority of HIV infected persons (more than 99%) are unable to control the virus in the absence of treatment. Even elite controllers continue to produce virus and suffer inflammatory consequences [4]. In HIV disease, the relative mitigating effects or associated features of antibody, cell-mediated or innate immune functions remain poorly understood. Since host immune responses to HIV are incapable of complete viral clearance, developing a vaccine to induce a successful immune response poses a particular challenge as no blueprint for vaccine design is provided by natural infection.

Another challenge to developing a successful vaccine against HIV is the nature of the virus. The viral genome within the human host is capable of profound and durable variability, particularly within the viral envelope gene that encodes the proteins most readily accessible by the immune system. In modeling studies, it is mutable enough to confound typical host development of broadly neutralizing antibody responses which require extensive somatic mutation in the immunoglobulin gene locus and obviation of immune tolerance mechanisms. [5] The virus is so adaptable that, in evading cytotoxic T-cell responses, multiple escape forms can appear simultaneously and persist rather than resulting necessarily in a single escape variant [6].

HIV, a retrovirus, genetically integrates into host chromosomes. There is genetic variation in the viruses that enter latency, the cells in which latency is established, how they become re-activated and how frequently the immune system responds to re-activation. Re-activation does not necessarily see the emergence of early, prevalent viral variants for which host immune responses would be more adapted to control, but could involve previously minor viral variants that have selective advantage over misdirected host immune responses [7, 8]. Recent evidence suggests that latency may be linked to how at least some HIV inter-variant recombinant viruses form. A latently infected cell can be super-infected with multiple viral variants, enabling the emergence of genetic recombinant forms in the re-activated viral quasi-species [9]. Cells housing re-activated HIV may present antigen infrequently and therefore be relatively insusceptible to antibody-dependent cellular cytotoxicity (ADCC) and other forms of immune surveillance [10].

The approach to developing a globally effective HIV vaccine has shifted over time. To date, there have been only six vaccine efficacy trials against HIV. They have included monomeric HIV envelope proteins alone and in combination with canarypox viral vectored HIV genes and adenoviral vectored HIV genes, alone or in combination with HIV DNA [11, 12]. They have not included HIV vaccines based on either viral attenuation or virion inactivation, for fully justified safety concerns, thus eliminating historically effective vaccine approaches for other pathogens. This has resulted in both a helpful orientation towards novelty and problematic new ground for how to begin.

## How

Despite these challenges, there now are reasons to be hopeful that an effective HIV vaccine is possible.

The RV144 HIV vaccine efficacy trial was conducted in over sixteen thousand men and women in Thailand randomized to receive either placebo or a combination of canarypox vector vaccine (ALVAC-HIV vCP1521) and an HIV glycoprotein 120 (gp120) recombinant product from subtypes B and E (AIDSVAX B/E) between 2003 and 2009. This remains the sole HIV vaccine trial for which protective efficacy was demonstrated. A modified intention to treat analysis which excluded those HIV infected at baseline observed a vaccine efficacy of 31.2% (95%CI, 1.1 to 52.1) 3 years after the primary vaccination series [13]. The protective effect six months following the primary vaccination series was 60.5% (95%CI, 22 to 80), but this effect waned quickly [14]. Vaccination influenced post-infection viral burden or CD4+ T-cell counts in participants with breakthrough HIV infection.

Immunologic markers associated with a protective effect of the vaccine have subsequently been identified. Through a broad collaboration of laboratories, multiple immune assays were studied from those volunteers who did and those who did not become infected with HIV. Through systematic down selection, primary assays were selected. The strongest association for protection were high IgG antibodies against the HIV envelope variable regions 1 and 2 (V1V2)—in particular IgG3—and low plasma IgA antibodies against envelope [15–17]. These results and continued work with RV144 and newer phase 2 studies with the RV 144 vaccine regimen have allowed progress in constructing improved assays and commonly applied study outcomes to measure success in a variety of settings and against multiple subtypes of HIV [18, 19].

RV144 proved a good opportunity to exploit and further develop systems biology approaches to correlates of risk of infection. A viral sieve analysis examining the genetic sequences of HIV in breakthrough infections, vaccine components and observed functional immune correlates helped both to elaborate associations and discern those which might be causal in protection [20]. For instance, antibody binding to the V1V2 region probably contributed to the protective effect while evidence was not present that vaccine induced T-cell responses did. These analyses also suggested that the vaccine itself did not contribute to mutations associated with breakthrough infection [21, 22]. Furthermore, different vaccine approaches confer different antibody-Fc complex profiles that may be important to protection through non-neutralizing effector functions [23]. Alter and colleagues demonstrated a strong correlation between ADCP and IgG1 subclass antibody against V1V2 using systems serology that was not otherwise discoverable with conventional associative approaches [23]. McElrath and colleagues similarly used a computational immunology approach to discover a novel T-cell phenotypic correlation with protection [24]. Exploratory trials following the RV144 population and others being conducted across large partner networks globally are mirroring these multilateral investigative approaches, as well as using tailored products against predominant circulating sub-types in other region, such as sub-type C in South Africa [25–27].

A non-vaccine oriented trial also has been illuminating. The story of how the immune system might partially or more fully control HIV is complex and, in some ways, has been told backwards, starting from the vantage of artificially induced immunity from vaccine trials rather than a true understanding of HIV infection as it establishes. After decades of experience with HIV patients, only recently has the acute course of infection started to be understood. Nearly 2300 high-risk patients in East Africa and Thailand were followed prospectively in a study called RV217. They experienced acute HIV infection at a rate of 5% [28]. Symptoms were absent in 71% of those who received physicals during the acute period, and neither symptoms nor signs were present in half. Peak viral load occurred at roughly 13 days, EIA positivity at 14 days and viral load set point at 31 days. Viral load set point was correlated with one-year outcomes. By the time of peak viral load—NK cells responded variably; B-cells had an initial decline then normalized; CD8+ T-cells increased; CD4+ T-cells decreased and remained inversely proportional to viral load. This information informs HIV vaccine strategy in several ways. HIV, like many other viral syndromes, completes the bulk of its distribution into host tissues in the first two weeks. It diversely impacts B and T cell populations with unclear impact on innate immunity. HIV causes less symptomatic acute disease (and by extension less system-wide inflammation) than previously thought. And, it stabilizes in an entrenched reservoir quickly. Consequently, neutralizing antibody available during logarithmic viral load expansion would be a useful but potentially insufficient mechanism for control. Acute inflammatory triggers cannot be relied upon from HIV to assist in activation of an amnestic response. Non-neutralizing antibody mediated—and possibly innate—effector functions will be important.

While consistent with hypotheses raised from RV144, and subsequent related work, application of lessons from RV 217 about which vaccine evoked host immune responses could be protective are speculative. They are aligned with current approaches to provide study participants exposures to HIV antigens in varied ways in order to elicit mixed immune responses from the humoral, cellular and innate arms of the host immune system using heterologous vaccine prime-boost approaches [17].

Much current effort in the field is directed at immunogen design that could evoke durable and broadly neutralizing antibody responses against HIV. The array of monoclonal antibodies capable of broadly neutralizing activity against HIV is growing exponentially owing to now prevalent molecular techniques to recover them from HIV infected patients. Some, such as PGT121, VRC01 and VRC03, have been shown in cellular assays to inhibit CD4+ T-cell entry by latent viruses derived from chronically infected persons [29]. Monoclonal antibody products of PGT121 and VRC01 administered to acutely infected rhesus macaques with hybrid Simian-Human Immunodeficiency Virus (SHIV) diminished both viral load and cell associated viral DNA (reservoir size) [30]. While the promise of evoking these response by active immunization remains elusive, their use for HIV prevention and control by passive immunization is being tested now in a range of clinical trials which would provide a proof of concept for why they would very likely work in active immunization approaches [31]. While early antibody identification relied only on empirically observed neutralization of various HIV strains by the sera of chronically infected or vaccinated individuals, the dawn of true antibody and antigen design is emerging. Researchers increasingly are becoming aware of the varied ways in which conformation, glycan shields and conserved zones within even highly variable regions play in antibody-antigen interactions in HIV infection [32–34].

## Conclusions

Insights from these various research efforts are myriad and critically important. They are advanced by clinical trial and observation, a fact that RV144 and its impact on HIV vaccine development illustrate. This requires continued investment in the capabilities that allow such work, including a durable framework to execute HIV vaccine trials in populations at risk for HIV infection. HIV vaccine research has become an innovating engine for vaccine research generally as demonstrated by the varied vaccine vector-derived products employed in trials for other pathogens that are grounded in HIV vaccine development. With continued focus, we will develop a globally effective HIV vaccine.
